# Purification of anti-glycoconjugate monoclonal antibodies using newly developed porous zirconia particles

**DOI:** 10.1038/s41598-021-82457-0

**Published:** 2021-02-09

**Authors:** Tetsuya Okuda, Katsuya Kato, Masahiro Kitamura, Shinjiro Kasahara

**Affiliations:** 1 Bioproduction Research Institute, National Institute of Advanced Industrial Science and Technology (AIST), Central 6, 1-1-1 Higashi, Tsukuba, Ibaraki 305-8566 Japan; 2 NGK Spark Plug-AIST Healthcare ・ Materials Cooperative Research Laboratory, National Institute of Advanced Industrial Science and Technology (AIST), 2266-98 Anagahora, Shimoshidami, Moriyama-ku, Nagoya, 463-8560 Japan; 3 NGK Spark Plug Co., Ltd., 2808 Iwasaki, Komaki, Aichi 485-8510 Japan

**Keywords:** Antibody isolation and purification, Protein purification, Glycobiology

## Abstract

Here, we describe porous zirconia particles (PZPs) optimized for the purification of immunoglobulins. PZPs, with a pore size of approximately 10 nm, were designed to specifically interact with antibodies via surface modification with a phosphate functional group. A simple PZP purification method based on precipitation enabled efficient purification of mouse anti-glycosphingolipid globoside/Gb4Cer monoclonal IgM (κ-light chains) from hybridoma culture supernatants. Over 99% of contaminating proteins were removed by the PZP purification process, and approximately 50% of the IgM was recovered in the purified fraction after eluting the PZP-adsorbed antibodies with 100 mM phosphate buffer. Other IgG3 and IgM monoclonal antibodies that react with Gb4Cer or α2,6-sialyl LacNAc-modified glycoproteins could also be purified using PZPs and elution buffer at concentrations of 100–500 mM. All of the purified antibodies retained their antigen reactivity and specificity, indicating that PZP purification does not affect antibody function. As PZP purification is also suitable for purification of IgM consisting of λ-light chains and IgG derived from other mammalian species, it is expected to be applied to the purification of a variety of antibodies, including anti-glycoconjugate IgMs.

## Introduction

The mammalian immune system recognizes carbohydrate antigens in a different manner than protein/peptide antigens^1–3^, which is related to the low induction of class switching. Thus, immunoglobulins (Igs) that recognize oligosaccharide epitopes in glycoconjugates such as glycolipids and glycoproteins are primarily produced as IgMs in mammals. Indeed, most monoclonal antibodies that recognize carbohydrate/oligosaccharide antigens with high reactivity and specificity are IgM^3–9^. In recent years, the application of IgMs as therapeutic agents and stem cell markers has been examined. For example, human IgMs that react with oligosaccharide epitopes of glycoproteins and glycolipids such as PAM-1, L612, and mAb216 are potential therapeutic or diagnostic agents for several cancers^10–12^. Major stem cell markers, such as SSEA-1 and SSEA-3, are also oligosaccharide antigens^13^, and IgMs against them have already contributed to stem cell technology. Thus, the development of materials/methods suitable for purifying IgM will contribute to the widespread practical use of anti-glycoconjugate antibodies.

Igs are generally purified using columns with immobilized Protein A or G, which specifically interact with the Fc-region of Igs^14,15^. However, these ligands do not interact with Igs of certain classes/subclasses or animal species due to low reactivity or structural issues^16^. In particular, these ligands are not suitable for the purification of IgM-class antibodies, as they cannot interact with the Fc-region of IgM molecules, which form an oligomeric structure^17^. A number of alternative methods have been investigated for the purification of IgM such as precipitation, ion exchange, size separation-based chromatography, and affinity purification^17^. However, none of them has become a general-purpose technology for IgM purification. The IgMs purified by these methods have basic performance problems such as poor purity and low biological activity. These methods have also run into problems with industrial applications such as cost, low stability and capacity, and the large number of steps for purification. These problems are also found in commercially available systems for IgM purification based on affinity-based chromatography using 2-mercaptopyridine, mannan-binding protein, anti-IgM, and protein L as ligands^17^. Thus, it is necessary to improve the current IgM purification technology for therapeutic and diagnostic applications of IgMs.

Previous model experiments using proteins such as bovine serum albumin (BSA) have demonstrated that porous zirconia particles (PZPs) can be used as adsorbents for proteins, and their application to chromatography for selective purification of certain proteins based on varying the pore size and chemically altering surface modifications has been proposed^18,19^. Clausen et al. reported that surface modification of zirconia by *N*,*N*,*N′*,*N′*-ethylenediaminetetrakis(methylenephosphonic acid) (EDTPA) enhances the selective interaction between zirconia microspheres and Igs^20^. The phosphate groups in EDTPA effectively modify the Lewis acids on the surface of zirconia and generate a unique selective interaction with Igs. It has also been reported that the EDTPA-modified zirconia microspheres can be used to separate Igs from hybridoma culture supernatants^21^ and human serum^22^. However, it remains unclear whether Igs purified by this zirconia maintain their reactivity to the antigen. In addition, as a general problem, zirconia particles show low protein adsorption capacity, which needs to be improved in order to utilize zirconia as a practical purification tool for Ig purification.

In this study, we newly developed PZPs with a pore size of approximately 10 nm that was designed to specifically interact with immunoglobulins via modification of the particle surface with EDTPA. These PZPs have a large specific surface area (133.46 m^2^/g) that allows interaction with a large amount of Igs, and the adsorbed Igs can be eluted by phosphate buffer (PB) at a neutral pH range, which is expected to maintain the biological activity of Igs. We examined the application of these PZPs to the purification of anti-glycoconjugate monoclonal antibodies (mAbs) from hybridoma culture supernatants.

## Results

### Development of PZPs suitable for antibody purification and a procedure for purifying monoclonal IgM

**Figure 1:**
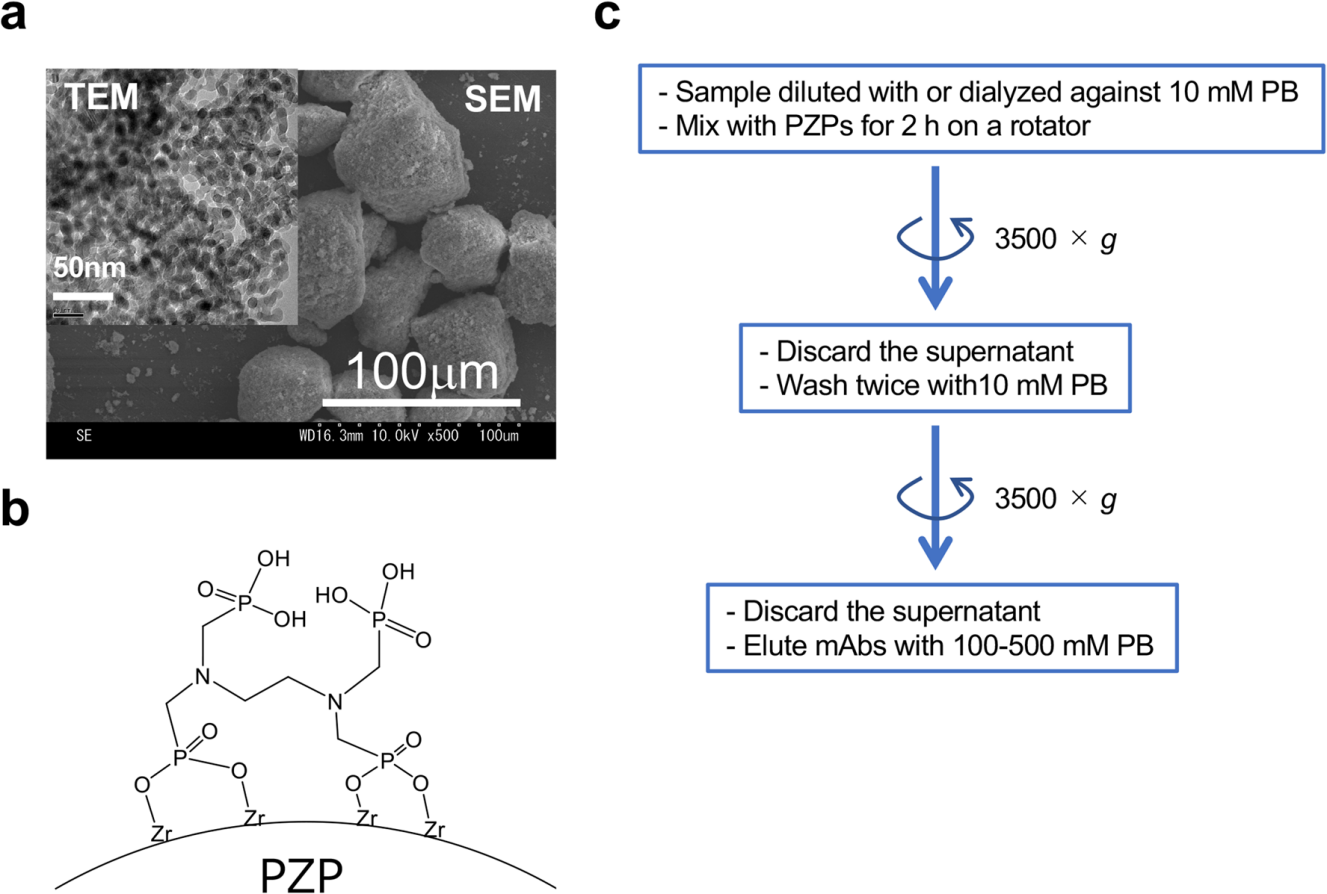
Electron microscope images of PZPs (**a**), chemical structure of the PZP surface (**b**), and overview of the procedure for Ig purification using PZPs (**c**).

PZPs with 10-nm pores and a surface modified with EDTPA (Fig. 1a,b) were prepared as described in the “Methods” section. The pore size was designed to be approximate to one Ig unit to facilitate selective interaction with Igs. In addition, the PZP surface was modified with EDTPA to improve selective interaction with Igs. The particle size aggregated was approximately 50 μm with a large surface area (133.46 m^2^/g), and the estimated Ig binding capacity of the PZPs was 50 mg/ml. The culture supernatant of hybridoma clone PA5, which produces mouse anti-Gb4Cer monoclonal IgM consisting of κ-light chains^8^ (Table 1), was used in this experiment. As the interaction between antibodies and PZPs was inhibited in culture medium with a high salt concentration, the supernatant was first dialyzed against 10 mM PB (pH 7.0), and the resultant concentrated sample was mixed with 20 mg of PZPs.

**Table 1:** Monoclonal antibodies used in this study.

Clone ID	Epitope (antigen glycoconjugate containing the epitope)	Class (Reference)
PA5	GalNAcβ1,3Galα1,4Galβ1,4Glc (Gb4Cer)	IgM(κ)^8^
PA4.2	GalNAcβ1,3Galα1,4Galβ1,4Glc (Gb4Cer)	IgG3(κ)^3,23^
PA7	GalNAcβ1,3Galα1,4Galβ1,4Glc or Galα1,4Galβ1,4Glc (Gb4Cer, Gb3Cer)	IgM(κ)^3^
FR9	Siaα2,6Galβ1,4GlcNAc (glycoproteins or glycolipids)	IgM(κ)^9^
MOPC104E	α1,3-Dextran (polysaccharides)	IgM(λ)^24^

**Figure 2:**
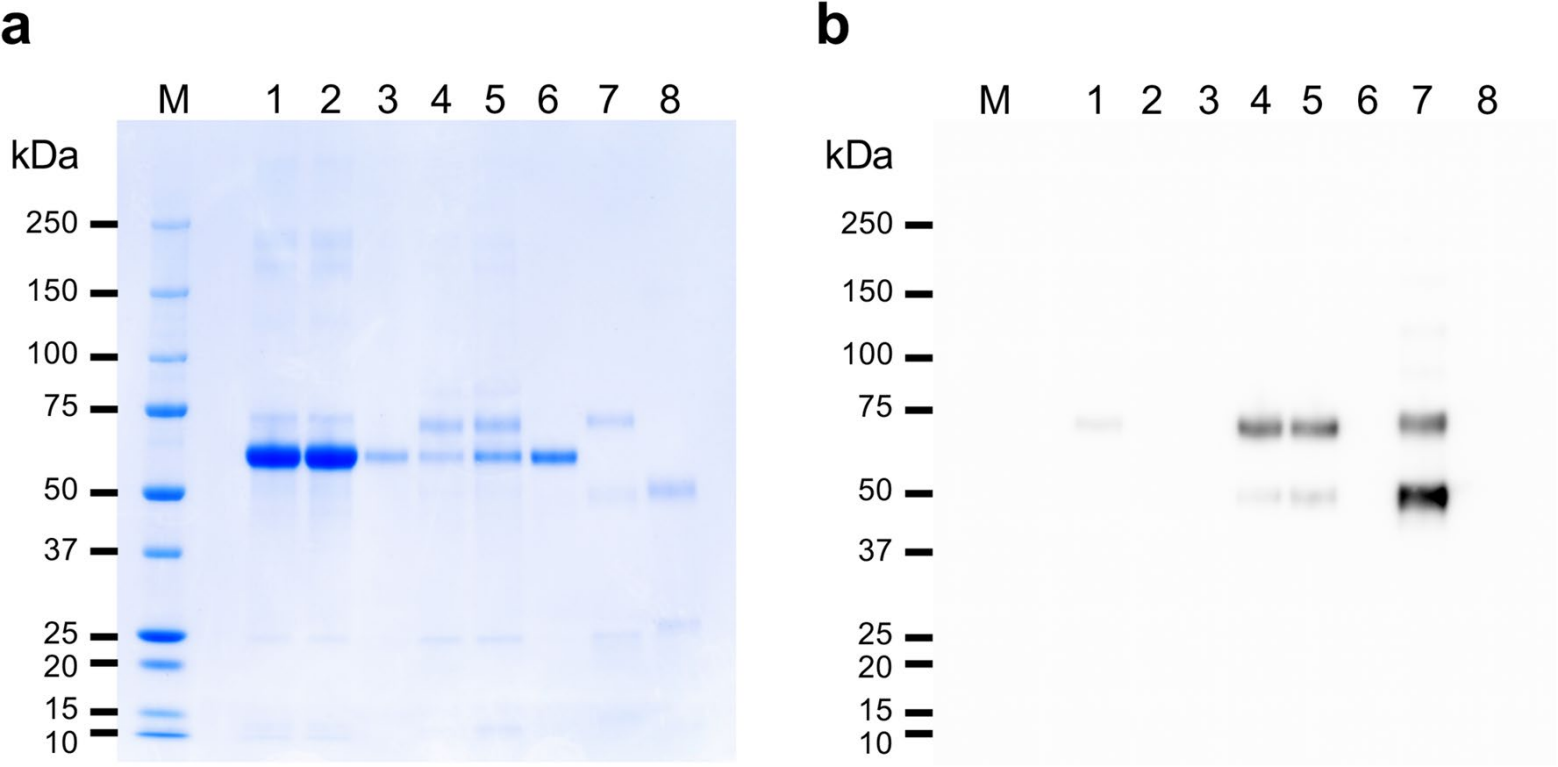
SDS-PAGE analysis of PA5 monoclonal IgM after PZP purification. Culture supernatants of hybridoma PA5 clone were purified using PZPs, and the following fractions were analyzed by SDS-PAGE with CBB staining (**a**) or anti-mouse IgM immunoblotting (**b**) as described in the “Methods”. Lane 1, hybridoma culture supernatant; 2, non-adsorbed fraction; 3, wash fraction; 4, 100 mM PB-eluted fraction; 5, 200 mM PB-eluted fraction; 6, standard BSA; 7, standard mouse IgM; 8, standard bovine IgG; M, molecular weight marker. The protein band neighboring the 50-kDa marker in lanes 4, 5, and 7 is a degradation product of the immunoglobulin μ-chain.

Ig purification using PZPs was based on precipitation (Fig. 1c), and the adsorbed antibodies were eluted from the PZPs using 10 × PB (100 mM, pH 8.0). Almost all of the antibodies were adsorbed by the PZPs, and over 50% of the antibodies were recovered in the elution fraction (Table 2a, 52.1%). SDS polyacrylamide gel electrophoresis (SDS-PAGE) indicated that although IgM was a minor protein in the original hybridoma culture supernatant containing 10% fetal bovine serum (FBS) (Fig. 2, lane 1), it was a major protein in the elution fraction (Fig. 2, lane 4). More than 99% of the contaminating proteins in the original sample were removed by the purification process, with the remaining contaminating protein mainly consisting of BSA. Another experiment using 200 mM PB (pH 8.0) as the elution buffer exhibited improved Ig recovery in the elution fraction (Table 2b, 59.2%; Fig. 2, lane 5). However, this condition also increased the amount of contaminating proteins and slightly decreased the purification rate (Table 2b, 20.8%).

**Table 2:** Amount of PA5 in each PZP-purified fraction.

Fraction	IgM (μg)	Recovery rate (%)	Total protein (mg)	IgM/total protein (%)
**(a) 100 mM elution**				
Culture sup	51.4	n/a	17.03	0.3
Non-adsorbed	0.7	1.7	n.d	n.d
Wash	0.5	1.0	n.d	n.d
Elution	26.8	52.1	0.07	38.3
**(b) 200 mM elution**				
Culture sup	52.7	n/a	17.03	0.3
Non-adsorbed	1.2	2.3	n.d	n.d
Wash	0.7	1.3	n.d	n.d
Elution	31.2	59.2	0.15	20.8

PA5 monoclonal antibodies in hybridoma culture supernatants were purified with PZPs using 100 mM (a) or 200 mM (b) phosphate buffer (pH 8.0) as the elution buffer.

*sup* supernatant, *n/a* not applicable, *n.d.* not determined.

### Application of PZP purification to monoclonal IgG3 and other IgM clones

To assess the applicability of the established procedure to other immunoglobulins, we purified several mAbs (Table 1) using PZPs (Table 3, Supplementary Fig. S1). In these experiments, we first examined the applicability of PZP purification to mouse anti-Gb4Cer monoclonal IgG3 produced by hybridoma clone PA4.2^3,23^. However, recovery of this IgG3 in the fraction eluted with 100 mM PB was poor. Sequential extraction of antibodies adsorbed to PZPs using 100 mM, 200 mM, and 500 mM PB (pH 8.0) revealed that recovery of this IgG3 was highest in the 200 mM PB elution fraction (Table 3a, 122.2 μg; Table 3b, 45.0%). We also examined the optimal elution buffer for PA7 and FR9 monoclonal IgMs^3,9^. PA7 is an anti-Gb4Cer IgM, but its antigen reactivity differs from that of PA5 (Table 1). FR9 reacts with α2,6-sialyl LacNAc residues in glycoproteins and glycolipids. The differing antigenic specificities of these IgMs indicate that the protein structure of the variable regions of PA5, PA7, and FR9 also differ. PA7 was eluted primarily in the 100 and 200 mM PB elution fractions (Table 3a, 21.4 and 21.5 μg), whereas FR9 was eluted primarily in the 200 mM and 500 mM PB elution fractions (Table 3a, 10.6 and 10.4 μg). Over 99% of the contaminating proteins in the respective supernatants were removed during the purification process for all antibodies, and approximately 50% of the antibodies were recovered in the main elution fractions (Table 3b).

**Table 3:** Amounts of PZP-purified antibodies in elution fractions.

Clone ID	Culture sup	100 mM	200 mM	500 mM	100–500 mM total
**(a) Total amount of antibody in each fraction (μg)**					
PA4.2	271.5	5.6	122.2	71.1	198.9
PA7	151.9	21.4	21.5	5.5	48.4
FR9	40.3	1.1	10.6	10.4	22.1
MOPC104E	60.8	40.9	10.4	7.1	58.4

Clone ID	100 mM	200 mM	500 mM	100–500 mM total	
**(b) Recovery rate of antibody in each fraction (%)**					
PA4.2	2.1	45.0	26.2	73.3	
PA7	14.1	14.1	3.6	31.9	
FR9	2.6	26.4	25.7	54.8	
MOPC104E	67.2	17.1	11.7	96.1	

Each antibody in hybridoma culture supernatant or ascites was purified using PZPs as described in the “Methods”, and antibodies adsorbed to PZPs were sequentially eluted using 100, 200, and 500 mM PB (pH 8.0). The total amount of each antibody (a) and the recovery rate from the original sample (b) in each elution fraction is summarized. SDS-PAGE and immunoblotting results for the antibodies in each fraction are shown in Supplementary Figs. S1 and S2.

### Effect of PZP purification on the antigen reactivity and specificity of antibodies

**Figure 3:**
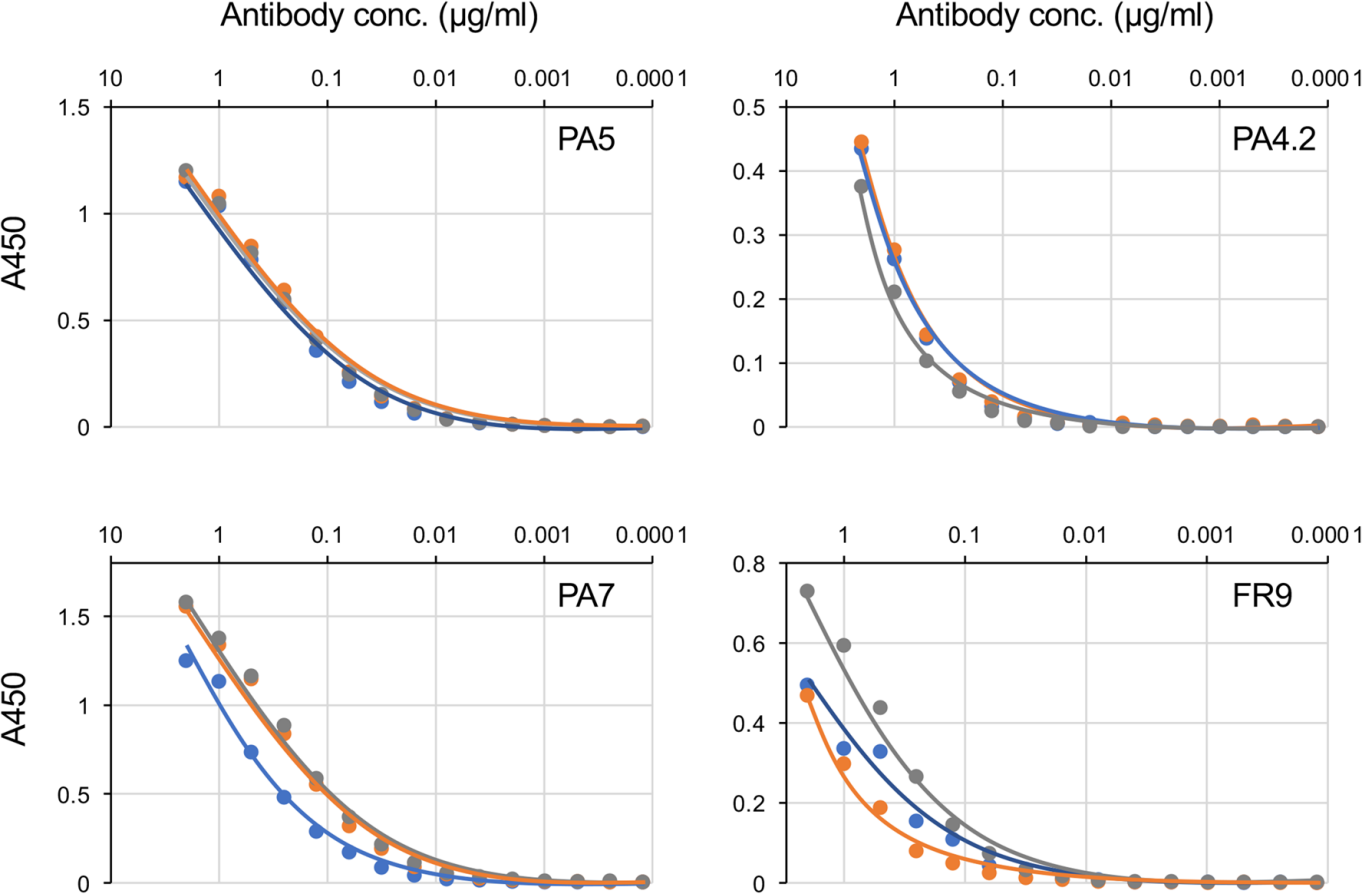
Analysis of the reactivity of PZP-purified antibodies with their respective antigens using ELISA. PZP-purified antibodies were serially diluted and reacted with their respective antigen, and the reactivity of each diluted antibody to the antigen was compared with that of the original hybridoma culture supernatant (blue line). The following main PZP elution fractions were analyzed for each antibody: 100 mM PB-eluted fraction (orange line in PA5 and PA7); 200 mM PB-eluted fraction (gray line in PA5 and PA7, orange line in PA4.2 and FR9); 500 mM PB-eluted fraction (gray line in PA4.2 and FR9). The following antigen-coated microplates were used in this analysis: for PA5, PA4.2, and PA7, 200 ng of Gb4Cer; for FR9, 1 μg of fetuin.

**Figure 4:**
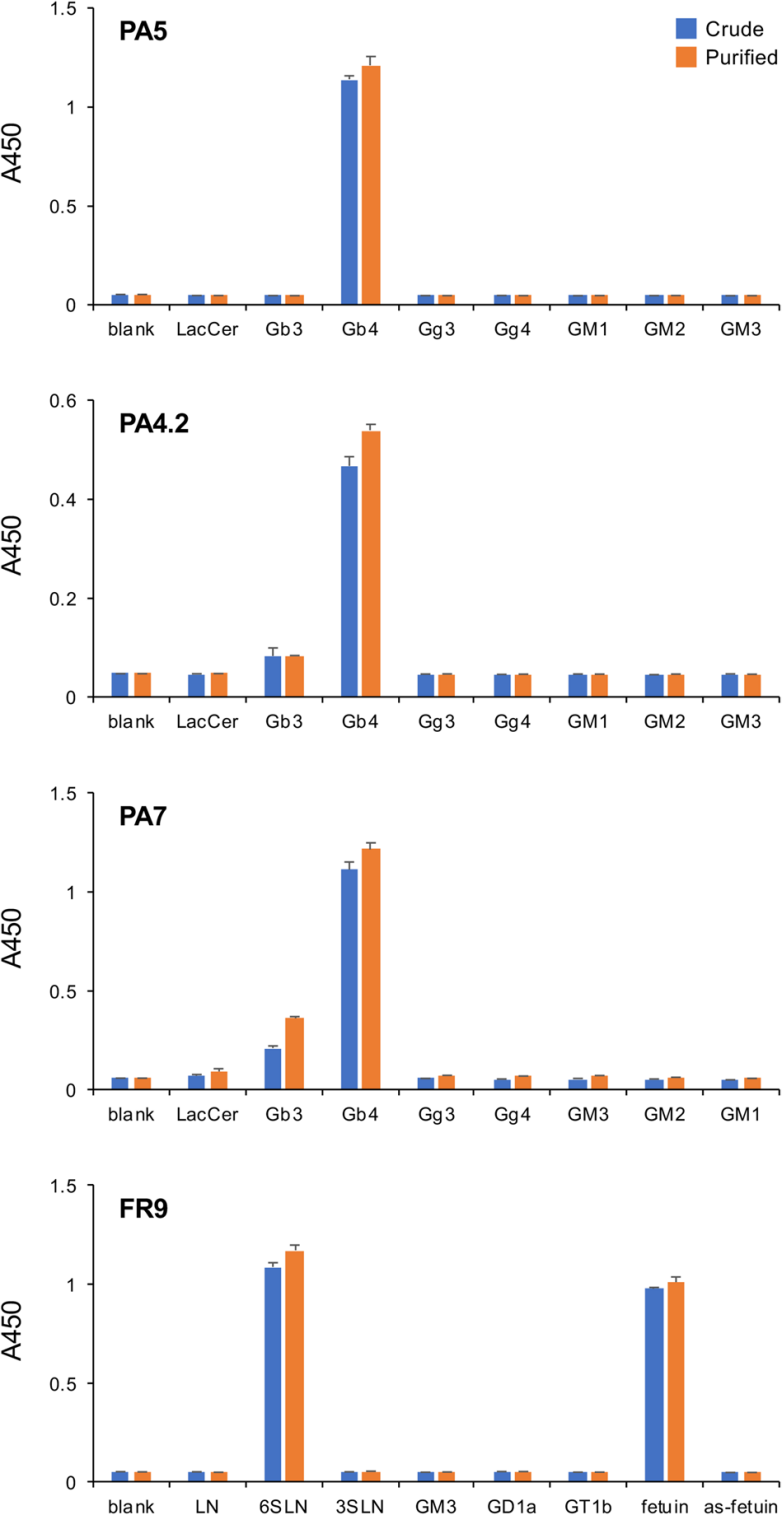
Analysis of the specificity of PZP-purified antibodies for their respective antigens using ELISA. PZP-purified antibodies (orange bars) were incubated in wells of microplates coated with the antigens or glycoconjugates similar to the antigens, and the reactivity of each antibody to the antigens was compared with that of the original hybridoma culture supernatant (blue bars). The following main PZP elution fractions were analyzed for each antibody: PA5, 100 mM PB-eluted fraction; PA4.2 and PA7, 200 mM PB-eluted fraction; FR9, 500 mM PB-eluted fraction. Error bars, mean ± S.D. *Gb3* Gb3Cer, *Gb4* Gb4Cer, *Gg3* Gg3Cer, *Gg4* Gg4Cer, *LN* LacNAcCerA, *6SLN* α2,6-sialyl LacNAcCerA, *3SLN* α2,3-sialyl LacNAcCerA, *as-fetuin* asialo-fetuin. The oligosaccharide structures of the glycoconjugates used in this analysis are shown in Supplementary Table S1.

To characterize the effect of PZP purification on various antibody properties, the reactivity of each PZP-purified Ig was analyzed by enzyme-linked immunosorbent assay (ELISA) (Fig. 3). Each Ig in its respective main elution fraction was serially diluted, and its antigen reactivity was compared with that of the Ig in the original supernatant sample. All of the PZP-purified antibodies retained antigen reactivity, and some of the antibodies exhibited increased antigen reactivity. An analysis using several glycoconjugates with structures similar to those of the antigens of these antibodies indicated that antigen specificity was also retained in the PZP purification procedure (Fig. 4). These results indicate that PZP purification has no adverse effects on the properties of antibodies and indeed may increase the reactivity of some antibodies to their antigen.

### Application of PZPs to the purification of IgM consisting of λ-light chains

All of the IgM antibodies used in this study consisted of κ-light chains, but the properties of the PZPs we developed suggested that they could be used to purify IgM(λ). To validate the applicability of PZPs to the purification to IgM(λ), we examined a commercially available mouse IgM consisting of λ-light chains (MOPC104E, Table 3, Supplementary Fig. S2)^24^. As this sample was prepared from ascites from a hybridoma-injected mouse, IgM constituted the main protein in the original preparation (Supplementary Fig. S2a, lane 1). PZPs adsorbed this IgM, and approximately 67% of the antibodies were recovered in the 100 mM PB elution fraction (Table 3a, 60.8 μg; Table 3b, 67.2%), indicating that PZP purification is also applicable to the purification of IgM(λ). Almost all of the IgM was recovered in the elution fraction (Table 3b, 96.1%).

### Effect of EDTPA modification and pores of PZPs on IgM purification

**Figure 5:**
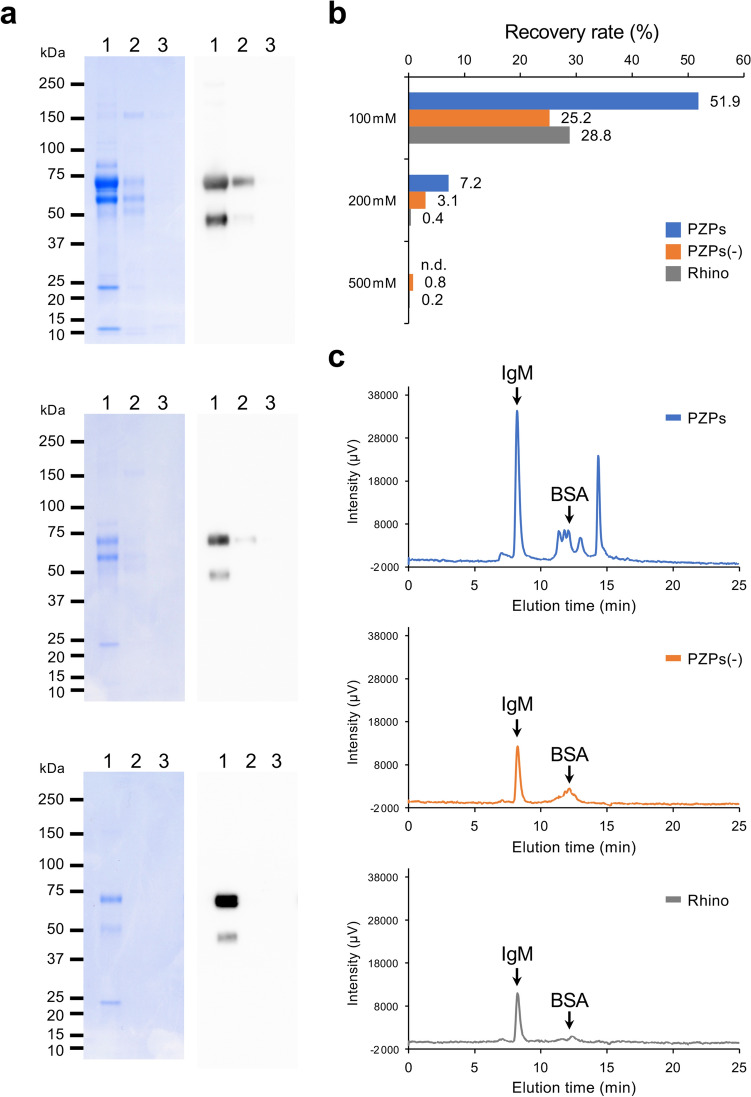
Effect of EDTPA modification of PZPs on Ig purification. (**a**) SDS-PAGE analysis of PA5 monoclonal IgM purified with PZPs (upper panel), EDTPA-unmodified PZPs (middle panel), and Rhinophase-AB (lower panel). The following PZP purification fractions were analyzed by SDS-PAGE with CBB staining (left panels) or anti-mouse IgM immunoblotting (right panels). Lane 1, 100 mM PB-eluted fraction; 2, 200 mM PB-eluted fraction; 3, 500 mM PB-eluted fraction. The protein band detected near the 50-kDa marker in lanes 4 and 8 is a degradation product of the immunoglobulin μ-chain. (**b**) The amount of Ig in each fraction was measured using IgM ELISA kits as described in the “Methods”, and the recovery rate of IgM from the original hybridoma culture supernatant was compared in each fraction. *PZPs* PZPs-purified sample, *PZPs(−)* EDTPA-unmodified PZPs-purified sample, *Rhino* Rhinophase-AB-purified sample. (**c**) The 100 mM eluted fractions were subsequently analyzed by SEC-HPLC. Upper panel, PZPs-purified sample; middle panel, PZPs(−)-purified sample; lower panel, Rhinophase-AB-purified sample. Arrows indicates the elution times of standard IgM and BSA as references.

To clarify the effect of modification of PZPs with EDTPA on IgM purification, IgM was purified using unmodified porous zirconia particles (PZPs(−)) from culture supernatants of hybridoma clone PA5 and compared with the PZPs-purified IgM. To examine the contaminating proteins, these purified samples were concentrated fourfold for use in subsequent analyses. SDS-PAGE showed that IgM and other proteins were efficiently eluted from PZPs(−) by 100 mM PB, similar to samples purified by PZPs (Fig. 5a, upper and middle panels). However, the signal intensities of all protein bands including IgM in the PZPs(−)-purified samples were lower than those in the PZPs-purified sample. The recovery rate of IgM in the main eluted fractions in the PZPs(−)-purified samples (Fig. 5b, 25.2%) was less than half that of the PZPs-purified samples (Fig. 5b, 51.9%). Size exclusion-high-performance liquid chromatography (SEC-HPLC) analysis showed that the percentages of IgM content out of total protein in the main 100 mM PB-eluted fractions were 46.5 and 50.0% in PZPs and PZPs(−)-purified samples, respectively (Fig. 5c). Furthermore, SEC-HPLC detected several unique protein peaks eluted around the BSA elution time only in the PZPs-purified samples. In particular, the small protein eluted at 14.1 min accounted for 23.2% of the total protein. As the percent of BSA among the total eluted protein was 6.9%, this indicates that certain proteins other than IgM in the culture supernatant were also specifically adsorbed on PZPs and eluted with 100 mM PB.

We also examined the purification of IgM from culture supernatants of hybridoma clone PA5 with previously reported EDTPA-modified zirconia particles (Rhinophase-AB)^20–22^. SDS-PAGE and SEC analysis showed that Rhinophase-AB could purify IgM with higher purity (84.3%) compared to PZPs and PZPs(−) (Fig. 5a,c, lower panels). However, the recovery rate of IgM in this eluted fraction was much lower (Fig. 5b, 28.8%) than for PZPs-purified samples. The specific surface areas calculated by the Brunauer–Emmett–Teller model for PZPs, PZPs(−), and Rhinophase-AB were 133.4576 m^2^/g, 131.2562 m^2^/g, and 9.8844 m^2^/g, respectively. These results indicated that the pores greatly expanded the specific surface area of PZPs and influenced their IgM binding capacity.

Taken together, modification of PZPs by EDTPA and pores efficiently improves the recovery rate of IgM, though it also increases the adsorption of certain non-Ig proteins that can be eluted with a mild PB.

## Discussion

In the first experiment, using mouse IgM PA5, which specifically reacts with the oligosaccharide in glycosphingolipid globoside/Gb4Cer^8^, over 99% of contaminating proteins were removed by a simple PZP purification procedure based on precipitation, and approximately 50% of the IgM was recovered in the 100 mM PB elution fraction. Other IgG3 or IgM mAbs that react with Gb4Cer^3,23^ or α2,6-sialyl LacNAc-modified glycoproteins^9^ could also be efficiently purified from the respective supernatants using the PZP procedure and eluting with PB in a range of 200–500 mM. All of the purified antibodies retained their antigen reactivity and specificity, indicating that PZP purification does not affect antibody function. Furthermore, PZPs could be also applied to the purification of IgM consisting of λ-light chains^24^ and IgG derived from other mammalian species. These results indicate that PZPs are suitable for the purification of any type of antibody, including anti-glycoconjugate IgM.

The established PZP purification system could be applied for the purification of several IgMs that react with different epitopes. These antibodies have structural differences in their variable regions, indicating that PZPs mainly interact with the constant regions of these IgMs. However, different concentrations of PB were required to elute the PZP-adsorbed Igs (Tables 2 and 3), which indicates that structural differences in IgMs also influence the strength of the PZP-IgM interaction. If PZP interacted with the Fc-region of Igs in the same manner as Proteins A and G, conformational constraints would preclude the purification of IgM using PZPs. Thus, PZPs interact with Igs in a different way from Protein A/G, which makes it possible for them to interact with various classes/subclasses of Igs. PZPs could also purify IgG molecules derived from FBS (Supplementary Fig. S3) that were contained in a small amount in hybridoma culture supernatants. These results indicate that PZPs are applicable to the purification of a variety of immunoglobulins without limit to particular classes/subclasses or animal species.

PZP-adsorbed antibodies can be eluted with PB, and the EDTPA modification of PZPs significantly improved the recovery rate of IgM (Fig. 5). These results indicate that the phosphate functional group modification of the PZP surface plays a role in the interaction with Igs. The main serum proteins such as BSA are acidic proteins that are electrostatically repulsive to phosphate groups. In contrast, Igs are basic proteins that have an electrostatic affinity with phosphate groups. In addition, the PZPs have pores with the same size as one unit of the Igs, which is expected to specifically enhance the interaction of PZPs with Igs. These pores also greatly expand the specific surface area of PZPs and contribute to their high IgM binding capacity. We conclude that the combination of these properties creates specific and effective interactions between Igs and PZPs.

The recovery rate of Igs purified using PZPs in the present study was approximately 50%, but almost no antibodies were detected in the non-adsorbed and wash fractions. This indicates that unrecovered antibodies remained adsorbed on the PZPs or were lost during sample preparation. As almost all of the antibodies in ascites samples could be recovered using PZP purification (Table 3b, 96.1%), this low recovery rate may be due to high concentrations of contaminating proteins and low concentrations of antibodies. Resolving these issues will improve the recovery rate.

As PZP purification can be performed using buffer in the neutral pH range, the antibodies are less adversely affected throughout the purification process. Indeed, the PZP-purified antibodies exhibited no decrease in either antigen reactivity or specificity (Figs. 3 and 4). Ig purification using Protein A or Protein G requires a strongly acidic buffer for elution, which frequently results in diminished antibody function^25^. Thus, PZPs are useful for purification because they preserve antibody function. Currently, several affinity resins consisting of Protein L^26^ or camelid antibodies specific to the κ- or λ-light chains are available for IgM purification. However, these ligands also require a strong acid to elute adsorbed Ig, which frequently leads to denaturation of the eluted Ig. As PZP is a simple inorganic material, it offers lower manufacturing costs than these protein ligands. This is also an advantage in scale-up.

Although many contaminating proteins in a sample can be removed by purification using Protein A or G, these contaminants cannot be completely removed in PZP purification. In the case of antibodies that require a high salt concentration for elution from PZPs in particular, the amount of contaminating proteins tends to be higher (Fig. 2, Table 2). The main contaminating protein in the PZP-purification fraction of hybridoma and ascites culture supernatants was BSA (Fig. 2 and Supplementary Fig. S2), which is generally used as a blocking agent for immunoassays. Thus, these contaminants are considered to have little impact on immunoassay applications, but in particular in pharmaceutical research and development, it would be necessary to remove these contaminants in Ig purification fractions. SEC-HPLC analysis indicates that other main contaminating proteins in PB-eluted fractions of PZPs are certain proteins that are only detected in the fraction purified by EDTPA-modified PZPs (Fig. 5c). Thus, inhibiting the interaction of PZPs with these proteins will increase the purity of Igs purified by the PZP. The results of this study suggest that these are basic proteins that interact with phosphate groups. Further analysis to identify these proteins is necessary to improve the purity of PZP-purified Igs and to develop PZP purification for pharmaceutical applications.

In the case of some anti-glycoconjugate antibodies examined in this study, PZP purification increased the antibody’s antigen reactivity (Fig. 3). Bovine serum contains a number of glycoproteins and glycolipids with the oligosaccharide epitope of these antibodies^27,28^ that results in inhibition of the antibody reactions. We speculate that PZP purification removes these glycoconjugates in hybridoma culture supernatants, resulting in an apparent increase in the reactivity of the purified antibodies to their antigens. As anti-glycoconjugate antibodies are predominantly of the IgM class, the PZP purification method established here is suitable for the preparation of these antibodies.

In conclusion, the developed PZP Ig purification method has unique characteristics that differ from currently used materials for antibody purification. These unique characteristics enable the purification of a variety of Igs, including those that cannot be purified using conventional approaches. PZPs can be used to purify antibodies without regard to animal species or Ig class/subclass, and it is expected that they will prove suitable for the purification of various industrial antibodies, such as antibody pharmaceuticals. The Igs adsorbed on PZPs can be eluted with PB in the neutral pH range. This is superior to prior technologies in maintaining of the function of purified Ig. Modification of PZPs by EDTPA and pores efficiently improves the recovery rate of IgM, though it also increases the adsorption of certain non-Ig proteins that can be eluted with a mild PB. Thus, further efforts are needed to improve PZP purification of Igs.

## Methods

### Materials

Zirconia particles used in this study were obtained from Nippon Denko Co., Ltd. (Tokyo, Japan). The surfaces of particles were modified by refluxing at 100 °C in 2.5 mM EDTPA aqueous solution (Tokyo Chemical Industry Co., Ltd., Tokyo, Japan). The determination of the specific surface area calculated from the nitrogen adsorption–desorption isotherms was conducted using TriStar II 3020 (Shimadzu Co., Kyoto, Japan) employing the Brunauer–Emmett–Teller model as described previously^29^. Rhinophase-AB was purchased from ZirChrom Separations, Inc. (Anoka, MN). Gb4Cer from human erythrocytes, fetuin from fetal calf serum, and IgM(λ) from murine myeloma (clone MOPC 104E, ascites fluid) were obtained from Sigma-Aldrich (St. Louis, MO, USA). Other glycoconjugate standards were obtained or prepared as described previously^9,23^. Mouse IgM and IgG3 standards were obtained from BioLegend (San Diego, CA) or prepared in-house. Albumin and IgG from bovine serum were obtained from Nacalai Tesque (Kyoto, Japan). Culture supernatants containing antibodies were prepared with hybridomas maintained in RPMI-1640 culture medium containing 10% FBS, 100 µM sodium hypoxanthine, 16 µM thymidine, 10 µg/mL gentamicin, and 5% Briclone (DS Pharma Biomedical, Osaka, Japan) at 37 °C in a humidified atmosphere containing 5% CO_2_.

### PZP purification of antibodies from hybridoma culture supernatants

Culture supernatants were dialyzed and concentrated against 10 mM PB (pH 7.0) using an Amicon Ultra-4 Ultracel-10K centrifugal device (Merck Millipore, Tullagreen, Carrigtwohill, Co., Cork, Ireland). PZPs were added to the dialyzed sample or PB-diluted ascites at a concentration of 20 mg/mL and mixed in a microtube using a rotator for 2 h. After centrifugation of the sample at 3500*g* for 2 min, the supernatant was collected (non-adsorbed fraction), and 10 mM PB was added to the PZPs at 20 mg/mL for washing. After mixing the PZPs with the wash buffer, the sample was centrifuged at 3500*g* for 2 min, the supernatant was removed and replaced with fresh wash buffer, and washing was repeated. Antibodies adsorbed onto the PZPs were eluted with 100–500 mM PB (pH 8.0) by mixing for 2 h, followed by centrifugation at 3500*g* for 2 min, after which the supernatant was collected as the elution fraction. The total volume of each fraction prepared in this study was as follows: hybridoma culture supernatant, 4 mL; dialyzed culture supernatant and non-adsorbed fractions, 1 mL; wash fraction, 2 mL (1 mL × 2); elution fraction, 0.2 mL. For SDS-PAGE, the following volumes were applied to the wells: hybridoma culture supernatant and PB-diluted ascites, 0.8 μL; non-adsorbed fractions, 0.2 μL; wash and elution fractions, 1 μL. The amount of Ig in each sample was measured using IgM or IgG3 ELISA kits (Thermo Fisher Scientific, Waltham, MA, USA) according to the manufacturer’s protocol.

### SDS-PAGE

Samples were heat denatured (98 °C, 3 min) under reducing conditions using 2-mercaptoethanol, and were separated by SDS-PAGE using SuperSep 5–20% gels (Wako, Osaka, Japan). The proteins in the gel were stained with Coomassie Brilliant Blue (CBB) or analyzed by immunoblotting as described previously^30^. For immunoblotting, protein bands in the gel were transferred onto an Immobilon-P PVDF membrane (Merck Millipore, Billerica, MA) by electroblotting at a constant current of 110 mA for 1 h. After blotting, the membrane was incubated with anti-mouse IgM (μ-chain specific) or IgG (γ-chain specific) peroxidase antibodies (0.2 μg/mL) obtained from Sigma-Aldrich. Antibody binding was detected using ECL Prime Western Blotting Detection Reagent and analyzed using ImageQuant LAS 500 software (GE Healthcare UK Ltd., Amersham, UK).

### ELISAs

ELISAs were performed as described previously^2^. In brief, glycolipids or glycoproteins were applied to the wells of a 96-well microtiter plate and incubated overnight. After washing twice with PBS, blocking buffer (1% BSA in PBS) was added to each well and incubated for 15 min at room temperature, followed by the addition of antibody. After incubation for 3 h at room temperature, the wells were washed with 0.1% Tween 20 in PBS, and then a horseradish peroxidase (HRP)-linked secondary antibody (anti-IgM or anti-IgG) was added. Antibody binding was detected using an HRP substrate (1-Step Ultra TMB-ELISA Substrate; Thermo Fisher Scientific) and measurement of absorbance at 450 nm. Samples were analyzed in duplicate in a single experiment.

### SEC-HPLC analysis

SEC-HPLC analysis of PZP purified samples was performed by a HPLC system (LC-2000, JASCO, Tokyo, Japan) using a TSKgel UltraSW Aggregate column (300 mm × 7.8 mm i.d., particle size 3 μm, Tosoh, Tokyo, Japan). Chromatographic analysis was carried out at 25 °C on the column using 0.2 M PB pH 6.7 as the mobile phase at a flow rate of 0.8 mL/min in isocratic mode. Igs and other proteins were detected by an in-line UV detector at 280 nm.

## Supplementary Information

Supplementary Information.

## Data Availability

The datasets generated during and/or analyzed during the current study are available from the corresponding author on reasonable request.
