# Supplementary material for: Purification of anti-glycoconjugate monoclonal antibodies using newly developed porous zirconia particles
Source: Sci Rep. 2021 Feb 9;11:3233. doi: 10.1038/s41598-021-82457-0 (PMC7873262; doi:10.1038/s41598-021-82457-0)
Supplement: Supplementary file 1 — Supplementary Information. [file 41598_2021_82457_MOESM1_ESM.pdf]

Purification of anti-glycoconjugate monoclonal antibodies using newly developed porous zirconia particles

Tetsuya Okuda[1*], Katsuya Kato[2],
Masahiro Kitamura[2] & Shinjiro Kasahara[3]

1. Bioproduction Research Institute, National Institute of Advanced Industrial Science and Technology (AIST), Central 6, 1-1-1 Higashi, Tsukuba 305-8566, Japan.

2. NGK Spark Plug-AIST Healthcare・Materials Cooperative Research Laboratory, National Institute of Advanced Industrial Science and Technology (AIST) , 2266-98 Anagahora, Shimoshidami, Moriyama-ku, Nagoya 463-8560, Japan

3. NGK Spark Plug Co., Ltd., 2808 Iwasaki, Komaki, Aichi 485-8510, Japan.

Supplementary Information

Supplemental Figure S1
Supplemental Figure S2
Supplemental Figure S3
Supplemental Table S1

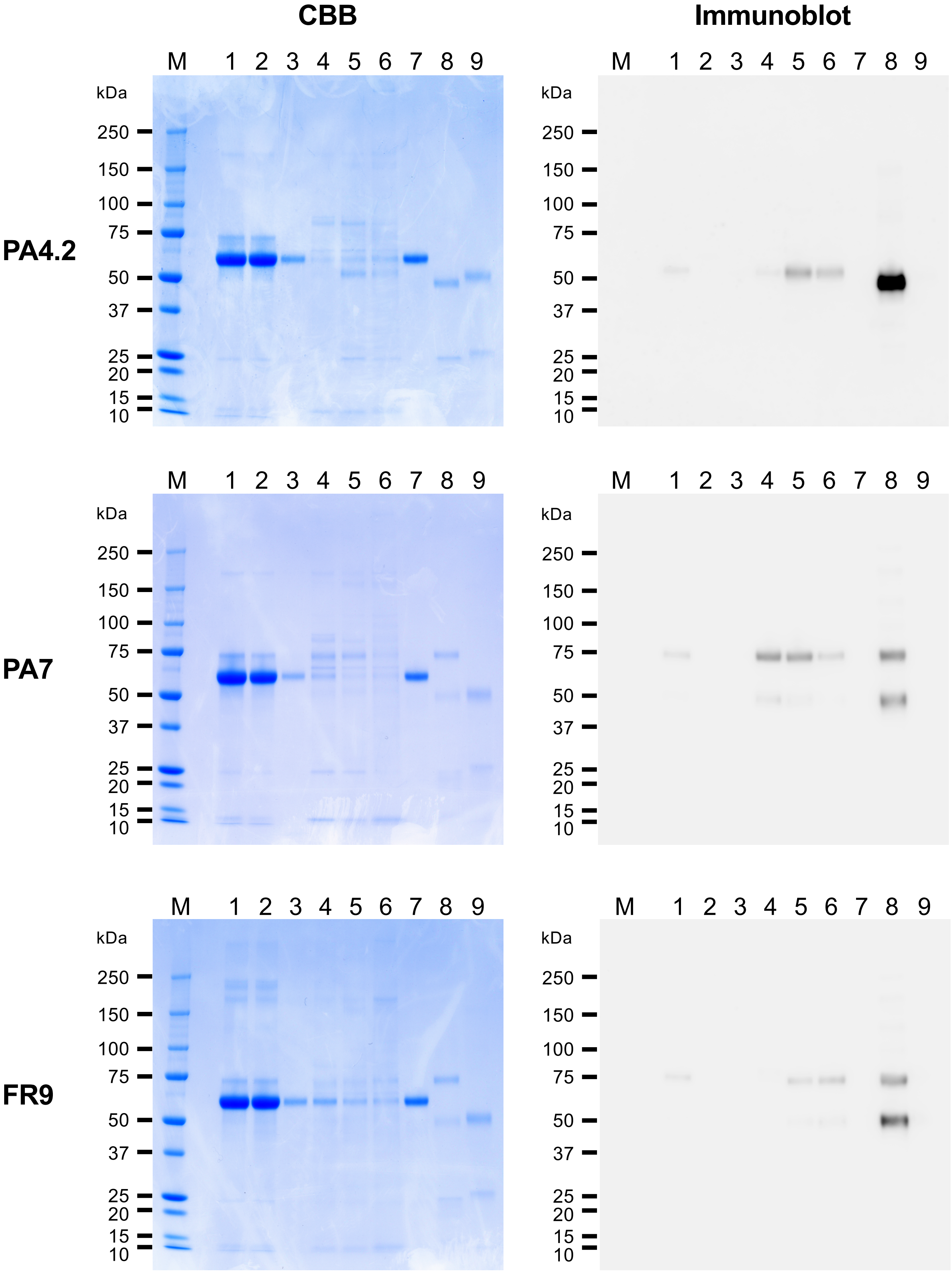

Figure S1. SDS-PAGE analysis of PZP-purified monoclonal antibodies.
Upper panels, PA4.2 IgG3; middle panels, PA7 IgM; bottom panels, FR9 IgM. Left panels, CBB staining; right panels, immunoblotting. Lane 1, hybridoma supernatant; 2, non-adsorbed fraction; 3, wash fraction; 4, 100 mM PB-eluted fraction; 5, 200 mM PB-eluted fraction; 6, 500 mM PB-eluted fraction; 7, standard BSA; 8, standard mouse IgG3 (for PA4.2) or IgM (for PA7 or FR9); 9, standard bovine IgG.

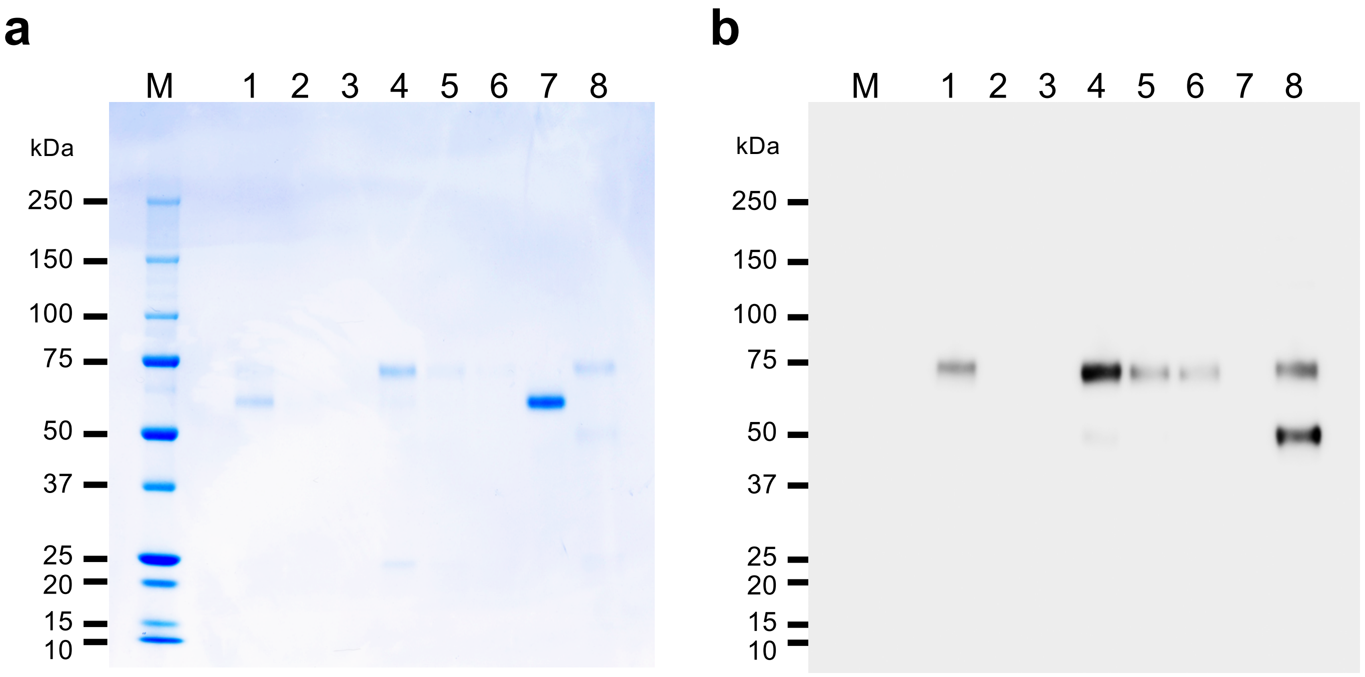

Figure S2. SDS-PAGE analysis of IgM composed by λ-light chains after PZP purification. Ascites fluid containing monoclonal IgM composed of λ-light chains (MOPC104E) was diluted with 10 mM PB (pH 7.0), and 1 mL of sample containing approximately 60 μg of these antibodies was purified using PZPs. The following PZP purification fractions were analyzed by SDS-PAGE with CBB staining **(a)** or anti-mouse IgM immunoblotting **(b)**, as described in the *Methods*. Lane 1, PB-diluted ascites; 2, non-adsorbed fraction; 3, wash fraction; 4, 100 mM PB-eluted fraction; 5, 200 mM PB-eluted fraction; 6, 500 mM PB-eluted fraction; 7, standard BSA; 8, standard mouse IgM; M, molecular weight marker. The protein band detected near the 50-kDa marker in lanes 4 and 8 is a degradation product of the immunoglobulin μ-chain.

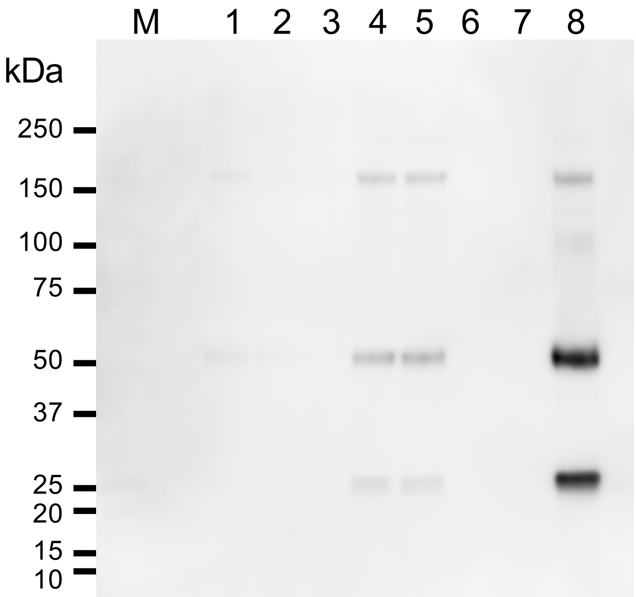

Figure S3. Anti-bovine IgG immunoblot analysis of PA5 purified from hybridoma supernatant using PZPs.

Bovine IgG in the SDS-PAGE sample (Fig. 2a) was detected by immunoblotting using HRP-labeled anti-bovine IgG as described in the *Methods*. Lane 1, hybridoma culture supernatant; 2, non-adsorbed fraction; 3, wash fraction; 4, 100 mM PB-eluted fraction; 5, 200 mM PB-eluted fraction; 6, standard BSA; 7, standard mouse IgM; 8, standard bovine IgG .

Glycoconjugate	Structure
LacCer	Galβ1,4GlcCer
Gb3Cer	Galα1,4Galβ1,4GlcCer
Gb4Cer	GalNAcβ1,3Galα1,4Galβ1,4GlcCer
Gg3Cer	GalNAcβ1,4Galβ1,4GlcCer
Gg4Cer	Galβ1,3GalNAcβ1,4Galβ1,4GlcCer
GM3	Siaα2,3Galβ1,4GlcCer
GM2	GalNAcβ1,4(Siaα2,3)Galβ1,4GlcCer
GM1	Galβ1,3GalNAcβ1,4(Siaα2,3)Galβ1,4GlcCer
GD1a	Siaα2,3Galβ1,3GalNAcβ1,4(Siaα2,3)Galβ1,4GlcCer
GT1b	Siaα2,3Galβ1,3GalNAcβ1,4(Siaα2,8Siaα2,3)Galβ1,4GlcCer
LacNAcCerA	Galβ1,4GlcNAcCerA
α2,6-sialyl LacNAcCerA	Siaα2,6Galβ1,4GlcNAcCerA
α2,3-sialyl LacNAcCerA	Siaα2,3Galβ1,4GlcNAcCerA
fetuin*	Siaα2,6Galβ1,4GlcNAc
asialo-fetuin*	Galβ1,4GlcNAc

Table S1. Structures of oligosaccharides in glycoconjugates used in this study. *Main structures of the non-reducing terminal oligosaccharides in these glycoproteins are presented. Abbreviations: Cer, ceramide; Sia, sialic acid (Neu5Ac); CerA, ceramide analogue.
